# Supplementary material for: Sublethal thermal stress promotes migration and invasion of thyroid cancer cells
Source: PLoS One. 2024 Feb 23;19(2):e0298903. doi: 10.1371/journal.pone.0298903 (PMC10889624; doi:10.1371/journal.pone.0298903)
Supplement: Attachment — Submitted filename: letter_R1.doc [file pone.0298903.s002.doc]

Dear Editor,

On behalf of all authors, I appreciate the time and effort of the editor and reviewers in critiquing our work (PONE-D-23-35946). The following are point-by-point responses to reviewers' comments. We believe that all the issues have been properly corrected and explained. This revised manuscript is being submitted for reconsideration for publication in *PLOS ONE*. Thank you for your great efforts.

Sincerely,

Shih-Ping Cheng, MD, PhD

Department of Surgery, MacKay Memorial Hospital

92, Chung-Shan North Road, Sec 2, Taipei 104217, Taiwan

Phone: +886 2 2543 3535, Fax: +886 2 2723 3897

E-mail: surg.mmh@gmail.com

**List of responses to comments**

Ms. No.: PONE-D-23-35946
Title: Sublethal thermal stress promotes migration and invasion of thyroid cancer cells

**For Editor**

**Comment:**

1. When submitting your revision, we need you to address these additional requirements.

Please ensure that your manuscript meets PLOS ONE's style requirements, including those for file naming. The PLOS ONE style templates can be found at

https://journals.plos.org/plosone/s/file?id=wjVg/PLOSOne_formatting_sample_main_body.pdf and

https://journals.plos.org/plosone/s/file?id=ba62/PLOSOne_formatting_sample_title_authors_affiliations.pdf

**Response:**

**We have edited the manuscript to fulfill these style requirements.**

**Comment:**

2. Thank you for stating in your Funding Statement:

This work was supported by research grants from the National Science and Technology Council of Taiwan (NSTC-111-2314-B-195-003 and NSTC-112-2314-B-195-009-MY3) and MacKay Memorial Hospital (MMH-11215 and MMH-11310). The funder had no role in study design, data collection and analysis, decision to publish, or preparation of the manuscript.

Please provide an amended statement that declares all the funding or sources of support (whether external or internal to your organization) received during this study, as detailed online in our guide for authors at http://journals.plos.org/plosone/s/submit-now. Please also include the statement "There was no additional external funding received for this study." in your updated Funding Statement.

Please include your amended Funding Statement within your cover letter. We will change the online submission form on your behalf.

**Response:**

**We moved the Funding Statement from the manuscript to the cover letter.**

**Comment:**

3. We note that your Data Availability Statement is currently as follows: All relevant data are within the paper.

Please confirm at this time whether or not your submission contains all raw data required to replicate the results of your study. Authors must share the "minimal data set" for their submission. PLOS defines the minimal data set to consist of the data required to replicate all study findings reported in the article, as well as related metadata and methods (https://journals.plos.org/plosone/s/data-availability#loc-minimal-data-set-definition).

**Response:**

**We uploaded our data set in EXCEL format as a supporting file.**

**Comment:**

4. Please review your reference list to ensure that it is complete and correct. If you have cited papers that have been retracted, please include the rationale for doing so in the manuscript text, or remove these references and replace them with relevant current references. Any changes to the reference list should be mentioned in the rebuttal letter that accompanies your revised manuscript. If you need to cite a retracted article, indicate the article's retracted status in the References list and also include a citation and full reference for the retraction notice.

**Response:**

**We revised the references to follow the journal's style.**

**For Reviewer #1:**

The research question is interesting and relevant to diverse thyroid cancer treatment trends. The authors tested the behavior of thyroid cancer cells in various temperatures and found the highest colony formation, migratory activity, and invasive capacity at 40 degrees. They suggest that sublethal thermal stress (which was 40 degrees) may increase clonogenicity, migration, and invasion of thyroid cancer cells. This information is vital for physicians choosing to apply RFA in thyroid cancer. However, several issues have to be clarified to make the deduction reasonable.

**Comment:**

1. The choice of three cell lines with different genetic backgrounds is commendable, but the rationale for selecting these specific cell lines is not provided in the introduction. Including a brief justification for choosing these particular cell lines would strengthen the rationale.

**Response:**

**We thank the reviewer for this comment. RFA as a treatment modality is confined to differentiated thyroid cancers, namely, papillary and follicular cancers/neoplasms. These cell lines were chosen to simulate clinical scenarios. Additionally, these cell lines are tumorigenic in immunocompromised mice based on our previous studies. This may facilitate subsequent animal studies.**

**Comment:**

2. The choice of a 10-minute heat treatment duration is explained based on the median RFA treatment time in clinical settings. However, based on the authors' information, the median treatment duration is around 15 minutes. Besides, the temperature range chosen for the heat treatment (up to 50 °C) should be discussed in the context of clinical relevance and safety. What is the potential range for a 40-degree in vivo? From the result, tumor cells are suppressed at 42 degrees or higher temperatures. Discussing how well these temperatures mimic the conditions encountered during RFA in thyroid cancer treatment would be beneficial.

**Response:**

**We thank the reviewer for this helpful suggestion. Previous studies showed that temperature distributions are affected by the distance and perfusion characteristics with wide variability. It is therefore difficult to estimate safe margins of ablation. We have expanded our discussion on this topic.**

**For Reviewer #2:**

Kuo and colleagues reported the first in vitro study in which thyroid cancer cells were subjected to sublethal heat stress for a transient time of 10 minutes. The authors found that migratory and invasive activity increased following heat treatment, accompanied by upregulation of epithelial-mesenchymal transition markers. The study has good scientific merit and a well-executed experimental design. The methods and statistical analysis appear appropriate. I have two minor comments on the study:

**Comment:**

1. It may be helpful to validate the findings of cellular studies in animal experiments. This is a weakness of this study.

**Response:**

**We thank the reviewer for pointing this out and have emphasized this weakness in our discussion.**

**Comment:**

2. Recently, a response-to-ablation system was proposed (doi: 10.1007/s00330-023-10022-6). Incomplete response is the main risk factor for local tumor progression. This information may be added to the article.

**Response:**

**We thank the reviewer for this suggestion and have included this article in the revised manuscript as reference #29.**
